# Pulmonary function in Thai patients with systemic sclerosis; a single center 6-year retrospective study

**DOI:** 10.12688/f1000research.146498.1

**Published:** 2024-04-18

**Authors:** Pattarin Pirompanich, Ornnicha Sathitakorn, Thitisak Sakulvorakitti

**Affiliations:** 1 Division of Pulmonary and Critical Care Medicine, Department of Medicine, Faculty of Medicine, Thammasat University, Bangkok, Bangkok, 12120, Thailand; 2 Department of Medicine, Faculty of Medicine, Thammasat University, Bangkok, Bangkok, 12120, Thailand

**Keywords:** Systemic sclerosis, Pulmonary function, Interstitial lung disease, ILD, Thailand

## Abstract

**Background:**

Interstitial lung fibrosis is a major cause of internal organ involvement and the leading cause of death in patients with systemic sclerosis (SSc). This study aimed to demonstrate the characteristics of pulmonary function (PF) in Thai patients with SSc and the association between PF and body mass index (BMI) and anti-topoisomerase (anti-Scl70).

**Methods:**

All patients diagnosed with SSc in our tertiary care teaching hospital database between 2016 and 2021 were reviewed. Clinical characteristics and PF were recorded and analyzed.

**Results:**

Of 211 SSc patients, 128 patients who underwent the PF test were enrolled; 102 (79.7%) were female. The mean (SD) age was 54.0 (12.5) years. The mean (SD) forced expiratory volume in one second (FEV1) forced vital capacity (FVC) ratio was 0.8 (0.1). The mean (SD) % predicted values of FEV1, FVC, and diffusing capacity of the lungs for carbon monoxide (DLCO) were 76.3 (16.3), 69.1 (15.8), and 75.5 (22.8), respectively. A restrictive spirometry pattern (RSP), defined as FVC < 80% predicted, was found in 78.8% of the patients. DLCO had a moderate positive linear correlation with FVC (r=0.50, p <0.001) and a moderate negative linear correlation with BMI (r=-0.36, p <0.001). However, there was no correlation between FVC and BMI. There was no statistical difference in demographic data or the presence of anti-Scl70 among patients with or without RSP.

**Conclusions:**

RSP is common among Thai patients with SSc. Spirometry is a cost-effective screening tool for detecting SSc-related pulmonary involvement in resource-limited settings. However, the power of using demographic data and the presence of anti-Scl70 to determine the probability of pulmonary complications remains limited.

## Background

Systemic sclerosis (SSc) is an autoimmune disease that results from microvascular damage, dysregulation of innate and adaptive immunity, and widespread fibrosis that affects multiple organs. While skin fibrosis is a key feature in patients with SSc, the clinical prognosis is determined by the severity of internal organ involvement. ^1^ The prevalence of SSc ranged from 38 to 341 cases per million, and the 5- and 10-year survival rates following diagnosis are 75% and 63%, respectively. ^2^

Major internal organ involvements in SSc include the pulmonary, cardiovascular, renal, and gastrointestinal systems. Notably, interstitial lung disease (ILD) has emerged as a primary complication during the initial stages in Thai patients and is a significant contributor to mortality. ^3^^–^^5^ The clinical presentations of SSc patients with ILD include dyspnea, non-productive cough, and fine crackles at the lung based on auscultation. The decline in forced vital capacity (FVC) was significantly higher in patients who had anti-topoisomerase autoantibody (anti-Scl70). In contrast, sex and age did not correlate with pulmonary function. ^3^

This study aimed to demonstrate the characteristics of pulmonary function in Thai patients with SSc and to explore the potential association between pulmonary function, body mass index (BMI), and the presence of anti-Scl70. The findings of this study may hold significant value in shaping management guidelines and provide insights for future studies on pulmonary complications in Thai patients with SSc.

## Methods

### Study design and setting

This was a single-center, 6-year retrospective observational study conducted between January 2016 and December 2021. This study was approved by the Human Research Ethics Committee of the Faculty of Medicine, Thammasat University, Thailand (Project number MTU-EC-IM-1-177/65, Approval number 193/2022, Date of approval September 19, 2022), which was conducted in accordance with the Declaration of Helsinki. The informed consent was waived in view of the retrospective nature of the study. Patient data were sourced from our institutional database at a 650-bed tertiary care university hospital.

### Patient selection and sample size

The enrollment criteria included individuals aged ≥ 18 years diagnosed with systemic sclerosis. All patients fulfilled the 1980 classification criteria for SSc ^6^ and underwent a pulmonary function test (PFT) during the study period. Demographic data, presence of anti-Scl70, forced expiratory volume in one second (FEV1), FVC, and diffusing capacity of the lungs for carbon monoxide (DLCO) were recorded. In cases with multiple PFT results for a single patient, the earliest test conducted during the study period was employed to mitigate the impact of ongoing treatment and disease progression. This retrospective study included all eligible patients with available data, providing a complete representation of the entire population under investigation.

### Outcomes

The primary objective was to identify the characteristics of pulmonary function in patients with SSc. The secondary outcomes included comparing the pulmonary function based on the presence of anti-Scl70, demonstrating the association between body mass index (BMI), FVC, and DLCO, and distinguishing characteristics of the patients who had restrictive spirometry patterns, defined as FVC of less than 80% predicted.

### Statistical analysis

Normality was assessed using the Shapiro-Wilk normality test. Categorical variables were reported as counts and percentages, and continuous variables as means with standard deviations (SD) or medians with interquartile ranges (IQR). Differences in continuous variables were compared using Student’s t-test or Mann-Whitney U test. Differences between separate groups of variables were compared using Fisher’s test or the chi-square test. The relationship between parameters was evaluated using Pearson’s correlation or Spearman’s correlation. Cases with any missing data points were removed from the analysis. A two-sided P value of less than 0.05 was considered to indicate statistical significance for the outcomes. Analyses were performed using STATA software 17.0 (StataCorp LLC, College Station, TX, US).

## Results

From a total of 211 SSc patients, 128 who underwent PFT were enrolled. Spirometry results were available for 118 (92.2%) patients, and DLCO results were available for 108 (84.4%) patients. The mean (SD) age of the patients was 54.0 (12.5) years, with 102 (79.7%) being female. The median (IQR) BMI for all patients was 21.7 (19.6-25.5) kg/m ^2^, as detailed in Table 1. ^9^

**Table 1.** Patient characteristics.

Characteristics	Overall (n = 128)
Age – mean (SD) years	54.0 (12.5)
Female – n (%)	102 (79.7)
BMI – median (IQR) kg/m ^2^	21.7 (19.6-25.5)
**Presence of anti-topoisomerase – n (%)**	
Positive	14 (10.9)
Negative	10 (7.8)
Not reported	104 (81.3)

BMI: body mass index; IQR: interquartile ranges; SD: standard deviations.

The primary outcome of the study revealed mean (SD) % predicted of FEV1, FVC, and DLCO as 76.3 (16.3), 69.1 (15.8), and 75.5 (22.8), respectively. The mean (SD) FEV1/FVC ratio was 0.8 (0.1). Notably, a restrictive spirometry pattern was predominant among the majority of the patients, accounting for 78.8% (93/118) of the study population. Further details regarding pulmonary function are presented in Table 2.

**Table 2.** Pulmonary function test of all participants.

Pulmonary function	Presence of spirometry (n = 118)
FVC – mean (SD) L	2.1 (0.6)
FVC – mean (SD) % predicted	69.1 (15.8)
FVC<80% predicted – n (%)	93 (78.8)
FEV1 – mean (SD) L	1.7 (0.5)
FEV1 – mean (SD) % predicted	76.3 (16.3)
FEV1/FVC ratio – mean (SD)	0.8 (0.1)
FEV1/FVC>0.75 – n (%)	102 (86.4)
	**Presence of DLCO (n = 108)**
DLCO – mean (SD) % predicted	75.5 (22.8)
DL adj – mean (SD) % predicted	78.5 (24.3)

FVC: forced vital capacity; FEV1: forced expiratory volume in one second; DLCO: diffusing capacity for carbon monoxide; DLadj: diffusing capacity for carbon monoxide adjusted for hemoglobin content.

**Figure 1.**
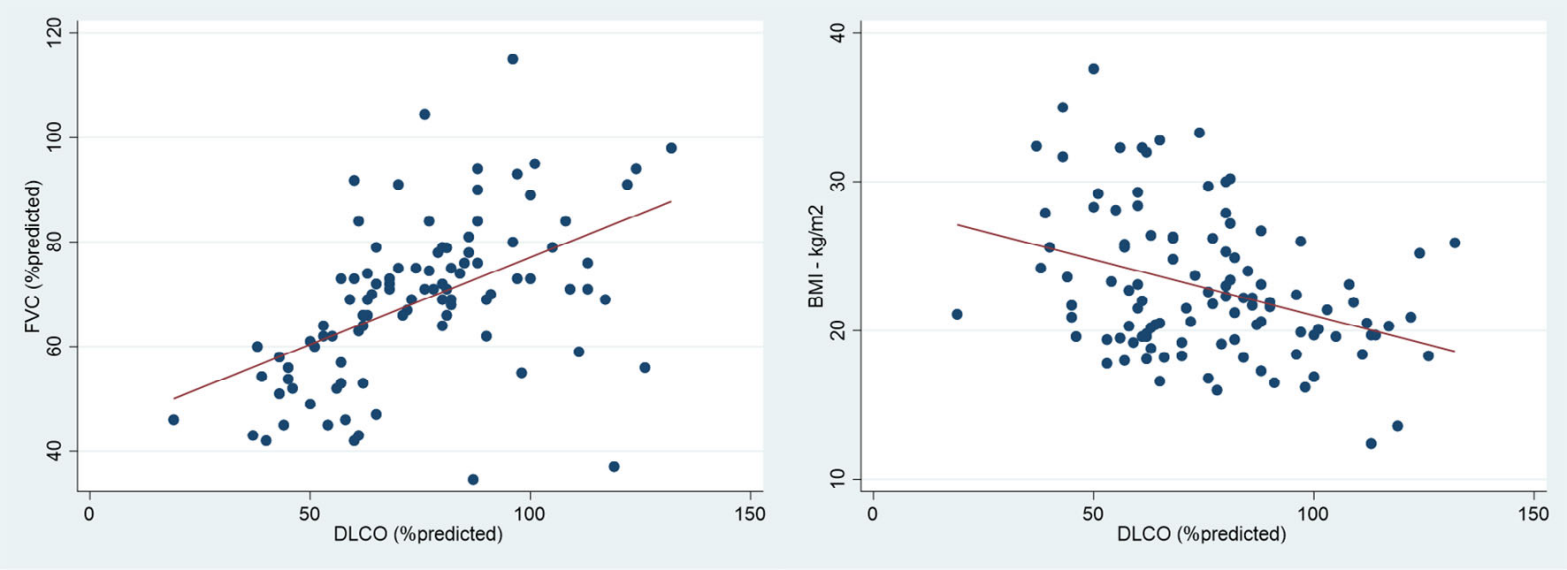
The correlation between diffusing capacity for carbon monoxide (DLCO) and forced vital capacity (FVC) (left) and DLCO and body mass index (BMI) (right).

In the secondary outcome analysis, following the categorization of patients based on the presence of anti-Scl70, no statistical differences were observed in FVC, FEV1, FEV1/FVC, or DLCO ( Table 3). DLCO demonstrated a moderate positive linear correlation with FVC (r=0.50, p<0.001) and a moderate negative linear correlation with BMI (r=-0.36, p<0.001) ( Figure 1). However, there was no correlation between FVC and BMI (r=-0.15, p-value=0.107).

**Table 3.** Pulmonary function test stratified by anti-topoisomerase antibody.

Pulmonary function	Anti-topoisomerase positive (n=14)	Anti-topoisomerase negative (n=10)	P-value
FVC – mean (SD) L	2.1 (0.5)	1.9 (0.7)	0.305
FVC – mean (SD) % predicted	68.4 (16.5)	71.4 (17.2)	0.673
FVC<80% predicted - n (%)	11 (78.6)	7 (70.0)	0.665
FEV1 – n (%)			
>70% predicted	6 (54.6)	2 (28.6)	
60-69% predicted	3 (27.3)	4 (57.1)	
50-59% predicted	1 (9.1)	0 (0.0)	
35-49% predicted	1 (9.1)	1 (14.3)	
<35% predicted	0 (0.0)	0 (0.0)	
FEV1 – mean (SD) L	1.8 (0.4)	1.5 (0.5)	0.203
FEV1 – mean (SD) % predicted	75.2 (18.0)	72.0 (15.7)	0.650
FEV1/FVC mean (SD)	0.8 (0.1)	0.8 (0.1)	0.203

	Anti-topoisomerase positive (n = 11)	Anti-topoisomerase negative (n = 7)	
DLCO – mean (SD) % predicted	64.5 (18.4)	70.3 (16.8)	0.509
DLCO – n (%)			
>75% predicted – n (%)	3 (27.3)	4 (57.1)	
61-75% predicted – n (%)	3 (27.3)	2 (28.6)	
40-60% predicted – n (%)	5 (45.5)	0 (0.0)	
<40% predicted – n (%)	0 (0.0)	1 (14.3)	
DLCO adj – mean (SD) % predicted	65.1 (19.0)	73.7 (16.9)	0.344

DLCO: diffusing capacity for carbon monoxide; DLadj: diffusing capacity for carbon monoxide adjusted for hemoglobin content; FEV1: forced expiratory volume in one second; FVC: forced vital capacity.

Patient characteristics, including age, sex, BMI, and the presence of anti-topoisomerase, did not demonstrate significant differences between those with and without a restrictive spirometry pattern. Notably, the majority of the patients with decreased DLCO exhibited a restrictive spirometry pattern ( Table 4).

**Table 4.** Patient characteristics stratified by restrictive spirometry pattern.

	FVC≥80% predicted (n=25)	FVC<80% predicted (n=93)	p-value
Age – mean (SD) years	53.6 (15.0)	54.7 (12.1)	0.688
Sex – n (%)			0.589
Female	21 (84.0)	72 (77.4)	
Male	4 (16.0)	21 (22.6)	
BMI – median (IQR) kg/m ^2^	21.7 (19.9-24.4)	21.8 (19.6-25.8)	0.489
Anti-topoisomerase – n (%)			0.665
Positive	3 (50.0)	11 (61.1)	
Negative	3 (50.0)	7 (38.9)	
DLCO – n (%)			0.018
>75% predicted	15 (83.3)	33 (41.3)	
61-75% predicted	2 (11.1)	21 (26.3)	
40-60% predicted	1 (5.6)	22 (27.5)	
<40% predicted	0 (0.0)	4 (5.0)	

BMI: body mass index; DLCO: diffusing capacity for carbon monoxide; FVC: forced vital capacity.

## Discussion

In this study, the mean % predicted values of FVC and DLCO were 69.1 and 75.5, respectively. A substantial majority of the patients (78.8%) exhibited a restrictive spirometry pattern. These results are consistent with those of a prior study on ILD in Thai patients with SSc, where the % predicted FVC ranged from 71.8 to 77.6. ^7^ In that study, the mean % predicted FVC and incidence of ILD within 5 years from disease onset were 71.8 and 86% in patients with diffuse cutaneous SSc, and 77.6 and 54% in patients with limited cutaneous SSc, respectively. ^7^ These findings emphasize the impact of pulmonary involvement on SSc.

However, the % predicted FVC in our study and the previously mentioned study appears to be lower than that reported in a previous large-scale study, The European Scleroderma Trials and Research group (EUSTAR) cohort, ^3^ where the mean % predicted FVC and DLCO in SSc patients were 93.5 and 68.9, respectively. This discrepancy may be attributed to the fact that the EUSTAR cohort assessed patients who presented within one year after the onset of Raynaud’s phenomenon, the most common initial presentation, accounting for 59.5% of SSc cases. ^5^ Furthermore, the EUSTAR cohort’s shorter disease duration compared to the referenced Thai study ^7^ might have contributed to the higher FVC values observed. These findings suggest a potential negative impact of disease duration on FVC, emphasizing the need for further studies to comprehensively explore this relationship.

Our study revealed no association between demographic data and restrictive spirometry results in patients with SSc. This suggests that the manifestation of a restrictive spirometry pattern in patients with SSc may be influenced by disease-specific factors rather than by general demographic characteristics. Similarly, the presence of anti-topoisomerase antibody did not significantly affect pulmonary function, although this finding diverges from previous studies suggesting a negative association with pulmonary outcome. ^3^^,^^7^ However, it is important to note that a majority of our patients in this study demonstrated a restrictive spirometry pattern and only a small number of our patients had documented records of the presence of this antibody. These limitations may constrain our ability to thoroughly assess the impact of these factors on the pulmonary function. Notably, reduced chest wall compliance, potentially contributing to restrictive pulmonary defects, could also be found in SSc, which may have influenced our results.

DLCO and FVC are the two most frequently used PFT for assessing the outcomes of SSc ILD. ^8^ SSc usually undergo regular DLCO and FVC. Our findings revealed a moderate linear correlation between DLCO and FVC, and nearly all patients with decreased DLCO exhibited a restrictive spirometry pattern. This finding suggests that spirometry can serve as an effective screening test for pulmonary involvement in SSc and is a cost-effective option, particularly in resource-limited settings.

In this study, some essential medical history, particularly patient symptoms, previous treatments, disease duration, and co-existing organ involvement, were documented in diverse formats, posing challenges for analysis. Therefore, the earliest test conducted during the study period was employed to mitigate the impact of ongoing treatment and disease progression. However, this limits our ability to evaluate the relationship between pulmonary function and these factors. Further studies with pre-specified data collection of these factors may reveal new tools for the detection of pulmonary involvement.

The strengths of our study are its considerable number of participants in a data-scarce field and being the only study that aims to explore pulmonary function exclusively in Thai patients with SSc. The effect of selective bias in the study is modest because most of our patients with SSc were screened with spirometry annually, regardless of patient symptoms.

However, this study has some limitations. First, since SSc is a progressive disease, lack of disease duration data limited us from defining patient disease stage and severity. Second, the small number of documented anti-topoisomerase antibodies decreased our power to examine its relationship with patient pulmonary function. Third, our study did not capture data on the type of systemic sclerosis in all participants. This omission limits our ability to assess the potential influence of SSc type on FVC and DLCO. Lastly, our study did not include pulmonary hypertension (PH) assessments, thus limiting our ability to conclusively determine the extent to which PH contributed to the observed DLCO values.

## Conclusion

A restrictive spirometry pattern is common among Thai patients with SSc. Spirometry is a cost-effective screening tool for detecting SSc-related pulmonary involvement in resource-limited settings. However, the power of using demographic data and presence of anti-topoisomerase to determine the probability of pulmonary complications remains limited. Further studies are required to evaluate anti-topoisomerase antibody data, SSc type, and pulmonary hypertension assessment.

## Ethics and consent

Ethical approval was obtained from the Human Research Ethics Committee of Thammasat University (Faculty of Medicine), Thailand (Project number MTU-EC-IM-1-177/65, Approval number 193/2022, Date of approval September 19, 2022), and the study was conducted according to the Declaration of Helsinki. The informed consent was waived in view of the retrospective nature of the study.

## Data Availability

Zenodo: Pulmonary function in Thai patients with systemic sclerosis data. https://zenodo.org/doi/10.5281/zenodo.10440509. ^9^ Data are available under the terms of the Creative Commons Attribution 4.0 International license (CC-BY 4.0).
