# Supplementary material for: Pulmonary function in Thai patients with systemic sclerosis; a single center 6-year retrospective study
Source: F1000Res. 2024 May 31;13:296. Originally published 2024 Apr 18. [Version 2] doi: 10.12688/f1000research.146498.2 (PMC11148527; doi:10.12688/f1000research.146498.2)
Supplement: Peer review history

# Reviewer report: Reviewer response for version 2

**DOI:** 10.5256/f1000research.166744.r285386

**Published:** 2024-06-04

**Authors:** Dujrath Somboonviboon

**Affiliations:** 1 Phramongkutklao Hospital, Bangkok, Thailand

I appreciate the author's prompt and thorough responses to my initial comments. The manuscript is now much clearer and all of my previous comments and suggestions have been effectively addressed.

 I have no further comments to make.

Is the work clearly and accurately presented and does it cite the current literature?

Partly

If applicable, is the statistical analysis and its interpretation appropriate?

Yes

Are all the source data underlying the results available to ensure full reproducibility?

No source data required

Is the study design appropriate and is the work technically sound?

Yes

Are the conclusions drawn adequately supported by the results?

Partly

Are sufficient details of methods and analysis provided to allow replication by others?

Partly

Reviewer Expertise:

Internal Medicine and Pulmonology.

I confirm that I have read this submission and believe that I have an appropriate level of expertise to confirm that it is of an acceptable scientific standard.

# Reviewer report: Reviewer response for version 2

**DOI:** 10.5256/f1000research.166744.r285384

**Published:** 2024-06-04

**Authors:** Pattraporn Tajarernmuang

**Affiliations:** 1 Division of Pulmonary, Critical Care, and Allergy, Department of Internal Medicine, Chiang Mai University, Chiang Mai, Thailand

I have no further comment.

Is the work clearly and accurately presented and does it cite the current literature?

Yes

If applicable, is the statistical analysis and its interpretation appropriate?

Yes

Are all the source data underlying the results available to ensure full reproducibility?

No source data required

Is the study design appropriate and is the work technically sound?

Yes

Are the conclusions drawn adequately supported by the results?

Yes

Are sufficient details of methods and analysis provided to allow replication by others?

Partly

Reviewer Expertise:

pulmonary, interventional respirology, lung cancer, critical care

I confirm that I have read this submission and believe that I have an appropriate level of expertise to confirm that it is of an acceptable scientific standard.

# Reviewer report: Reviewer response for version 2

**DOI:** 10.5256/f1000research.166744.r285385

**Published:** 2024-06-03

**Authors:** Chingching Foocharoen

**Affiliations:** 1 Department of Medicine, Khon Kaen University, Nai Mueang, Khon Kaen, Thailand

The manuscript has been revised with more clarification compared to the previous version. However, I still have some points of concern below:

1. The low numbers of results reporting anti-Scl70 and the lack of fluctuation in anti-Scl70 levels may not be appropriate to evaluate the association between the presence of anti-Scl70 and PFT. I suggest evaluating the level of anti-Scl70 instead of the presence or absence of anti-Scl70.
2. Longitudinal evaluation of the follow-up of anti-Scl70 and changes in PFT may not be advisable for further research because seroconversion is rarely observed in SSc patients. (Ref- 1)

Is the work clearly and accurately presented and does it cite the current literature?

Partly

If applicable, is the statistical analysis and its interpretation appropriate?

Yes

Are all the source data underlying the results available to ensure full reproducibility?

No source data required

Is the study design appropriate and is the work technically sound?

Yes

Are the conclusions drawn adequately supported by the results?

Partly

Are sufficient details of methods and analysis provided to allow replication by others?

Partly

Reviewer Expertise:

Internal Medicine and Rheumatology

I confirm that I have read this submission and believe that I have an appropriate level of expertise to confirm that it is of an acceptable scientific standard.

## References

[1] : Incidence of anti-topoisomerase I antibody seroconversion and clinical association in systemic sclerosis. *Asia-Pacific Journal of Science and Technology, 26(02), APST–26. https://doi.org/10.14456/apst.2021.24* .2021;

# Reviewer report: Reviewer response for version 1

**DOI:** 10.5256/f1000research.160591.r271795

**Published:** 2024-05-23

**Authors:** Pattraporn Tajarernmuang

**Affiliations:** 1 Division of Pulmonary, Critical Care, and Allergy, Department of Internal Medicine, Chiang Mai University, Chiang Mai, Thailand

Thank you for the invitation to review the manuscript by Pirompanich et al. The authors presented a single-center retrospective study aiming to identify the clinical characteristics of pulmonary function in patients with systemic sclerosis. The minor concerns and suggestions for this study are as follows:

1. As we know, lung involvement in diffuse systemic sclerosis (SSc) is more common than in limited SSc. It would be beneficial to demonstrate the proportion of diffuse SSc and limited SSc in the patient characteristics.
2. There were 211 SSc patients, but only 128 (~60%) patients who had PFT results were enrolled. The possible reason for the unavailability of PFT results might be that the patients were too sick to perform the test. This selection bias could make the PFT results appear too favorable. The authors should mention this point in the limitations of the study.
3. The authors mentioned that limited chest wall compliance can cause restrictive pulmonary defects; however, lung imaging (e.g., HRCT) can help distinguish between chest wall and lung problems. Also, some patients with abnormal HRCT can have normal spirometry. I think the authors should clarify this point.

Is the work clearly and accurately presented and does it cite the current literature?

Yes

If applicable, is the statistical analysis and its interpretation appropriate?

Yes

Are all the source data underlying the results available to ensure full reproducibility?

No source data required

Is the study design appropriate and is the work technically sound?

Yes

Are the conclusions drawn adequately supported by the results?

Yes

Are sufficient details of methods and analysis provided to allow replication by others?

Partly

Reviewer Expertise:

pulmonary, interventional respirology, lung cancer, critical care

I confirm that I have read this submission and believe that I have an appropriate level of expertise to confirm that it is of an acceptable scientific standard, however I have significant reservations, as outlined above.

# Response

**Published:** 2024-05-27

**Authors:** Pattarin Pirompanich

**Affiliations:** Medicine, Thammasat University, Klong Luang, Pathumthani, Thailand

**Response to reviewer** **3:**

 Thank you very much for you kindly review. We really appreciate your response and revised our manuscript as you suggested.

**Point 1:** As we know, lung involvement in diffuse systemic sclerosis (SSc) is more common than in limited SSc. It would be beneficial to demonstrate the proportion of diffuse SSc and limited SSc in the patient characteristics.

**Response 1: ** We have acknowledged these on Discussion (Page 7, paragraph 2, line 8-10) and limitation (Page 8, paragraph 5, line 6-7).

**Point** **2:** There were 211 SSc patients, but only 128 (~60%) patients who had PFT results were enrolled. The possible reason for the unavailability of PFT results might be that the patients were too sick to perform the test. This selection bias could make the PFT results appear too favorable. The authors should mention this point in the limitations of the study.

**Response** **2: ** We have acknowledged these on limitation (Page 8, paragraph 5, line 10-12).

**Point** **3:** The authors mentioned that limited chest wall compliance can cause restrictive pulmonary defects; however, lung imaging (e.g., HRCT) can help distinguish between chest wall and lung problems. Also, some patients with abnormal HRCT can have normal spirometry. I think the authors should clarify this point.

**Response** **3: ** Thank you for the insightful comment regarding the role of chest wall compliance in restrictive lung disease. We acknowledge that reduced chest wall compliance can be a contributing factor, and ideally, lung imaging techniques like HRCT would be incorporated to differentiate between restrictive etiologies. Unfortunately, due to limitations in our study design, we were unable to collect data on chest wall compliance. Therefore, we have decided to remove the sentence mentioning this factor from the manuscript.

# Reviewer report: Reviewer response for version 1

**DOI:** 10.5256/f1000research.160591.r271791

**Published:** 2024-05-20

**Authors:** Dujrath Somboonviboon

**Affiliations:** 1 Phramongkutklao Hospital, Bangkok, Thailand

I appreciate the opportunity to review this article, which presents on pulmonary function in Thai systemic sclerosis patients, and I would like to offer a few comments and suggestions to further enhances its clarity and impact.

 Abstract

- The authors did not compare spirometry to other interventions, particularly HRCT. Therefore, the conclusion regarding the cost-effectiveness of spirometry should be revised.

Full manuscript 

 Background

- The correlation between pulmonary function and SSc is understandable, but the authors should explain why they were interested in BMI.  
- The authors mentioned the importance of ILD in SSc patients, however, this study did not include HRCT data, only pulmonary functions were recorded. Therefore, using ‘pulmonary involvement’ would be more appropriate than ILD.

Discussion

- There are several confusing points in the discussion section, particularly when the authors compare the findings from this cohort to those of previous studies.
- Please check the numbers, particularly regarding the data from the referenced papers.
- The authors found that FVC in this cohort was lower than in the EUSTAR cohort and explained this by noting that EUSTAR cohort had shorter disease duration. However, a previous Thai cohort [Ref -1] demonstrated that a shorter duration of pulmonary fibrosis after onset also correlated with poorer outcome based on the longitudinal data analysis. Furthermore, the mean disease duration in the referenced Thai study [Ref - 2] was only 12.9 months. Therefore, this part should be revised.
- As mentioned earlier, the authors did not investigate the cost-effectiveness of spirometry.

Is the work clearly and accurately presented and does it cite the current literature?

Partly

If applicable, is the statistical analysis and its interpretation appropriate?

Yes

Are all the source data underlying the results available to ensure full reproducibility?

No source data required

Is the study design appropriate and is the work technically sound?

Yes

Are the conclusions drawn adequately supported by the results?

Partly

Are sufficient details of methods and analysis provided to allow replication by others?

Partly

Reviewer Expertise:

Internal Medicine and Pulmonology.

I confirm that I have read this submission and believe that I have an appropriate level of expertise to confirm that it is of an acceptable scientific standard, however I have significant reservations, as outlined above.

## References

[1] : Clinical characteristics and outcomes of 566 Thais with systemic sclerosis: A cohort study. *Int J Rheum Dis* .2020;23(7) : 10.1111/1756-185X.13859 945-957 10.1111/1756-185X.13859 32420701

[2] : Incidence and predictors of interstitial lung disease (ILD) in Thai patients with early systemic sclerosis: Inception cohort study. *Mod Rheumatol* .2016;26(4) : 10.3109/14397595.2015.1115455 588-93 10.3109/14397595.2015.1115455 26561397

# Response

**Published:** 2024-05-27

**Authors:** Pattarin Pirompanich

**Affiliations:** Medicine, Thammasat University, Klong Luang, Pathumthani, Thailand

Thank you very much for you kindly review. We really appreciate your response and revised our manuscript as you suggested.

**Abstract**

**Point 1:** The authors did not compare spirometry to other interventions, particularly HRCT. Therefore, the conclusion regarding the cost-effectiveness of spirometry should be revised.

**Response 1: ** We have deleted this sentence from the abstract section and also in conclusion section.

**Background**

**Point 1:** The correlation between pulmonary function and SSc is understandable, but the authors should explain why they were interested in BMI.  

**Response 1: ** BMI was included because it's a readily available measure of body composition that might influence lung function through various mechanisms. Understanding this relationship can provide insights into disease severity and potential risk factors for SSc patients.

 We have added this data on Background (Page 2, paragraph 2, line 8-9) (reference 6).

- Ishikawa C, Barbieri MA, Bettiol H, Bazo G, Ferraro AA, Vianna EO. Comparison of body composition parameters in the study of the association between body composition and pulmonary function. BMC Pulm Med. 2021;21(1):178.

**Point** **2:** The authors mentioned the importance of ILD in SSc patients, however, this study did not include HRCT data, only pulmonary functions were recorded. Therefore, using ‘pulmonary involvement’ would be more appropriate than ILD.

**Response** **2: ** We have changed accordingly.

**Discussion**

**Point 1:** There are several confusing points in the discussion section, particularly when the authors compare the findings from this cohort to those of previous studies.

**Response 1: ** We have revised the data in the Discussion section to provide a clearer picture of the findings (Page 7, paragraph 1 and 2).

**Point** **2:** Please check the numbers, particularly regarding the data from the referenced papers.

**Response** **2: ** We have revised the data in the Discussion section to accurately reflect the information from the referenced papers (Page 7, paragraph 1 and 2).

**Point** **3:** The authors found that FVC in this cohort was lower than in the EUSTAR cohort and explained this by noting that EUSTAR cohort had shorter disease duration. However, a previous Thai cohort [Ref -1] demonstrated that a shorter duration of pulmonary fibrosis after onset also correlated with poorer outcome based on the longitudinal data analysis. Furthermore, the mean disease duration in the referenced Thai study [Ref - 2] was only 12.9 months. Therefore, this part should be revised.

**Response 3: ** We have revised the data in the Discussion section to provide a clearer picture of the findings and to accurately reflect the information from the referenced papers (Page 7, paragraph 1 and 2).

**Point** **4:** As mentioned earlier, the authors did not investigate the cost-effectiveness of spirometry.

**Response** **4: ** We have deleted this sentence from the abstract section and also in conclusion section.

# Reviewer report: Reviewer response for version 1

**DOI:** 10.5256/f1000research.160591.r271788

**Published:** 2024-05-10

**Authors:** Chingching Foocharoen

**Affiliations:** 1 Department of Medicine, Khon Kaen University, Nai Mueang, Khon Kaen, Thailand

The authors presented an association factors with pulmonary function in scleroderma. However, I believe there are some areas where additional details would strengthen the manuscript and provide a more convincing argument for the coexistence of both conditions. I have listed my points of concern below:

 Abstract

1. Mean BMI and the proportion of patient with positive for anti-Scl70 should be mentioned in abstract because they were endpoints of the study.
2. This study did not investigate the cost-effectiveness of spirometry. Please revise the conclusion in abstract section and also in conclusion section.

Background

1. The authors should mention why BMI was in your interested to be an endpoint of the study. What is/are the rationales for defining the association between BMI and spirometry parameters?

Methods

1. The diagnosis of systemic sclerosis is now using the 2013 ACR/EULAR Classification criteria not from 1980 ACR criteria.
2. 128 out of 211 SSc patients underwent spirometry, so nearly 40% were not performed the spirometry. This may cause selection bias. Of those 40% who were not performed the test, they might be too tired to do the test or have difficulty to do the test for example air leak from mouth related to skin tightness of face, etc. Please clarify this point.
3. Spirometry parameters and BMI can dynamically change or fluctuate over the course of follow-up, but anti-Scl70 is still be stable. Please clarify when of the spirometry test as well as BMI was picked up for analysis.
4. The association between spirometry and anti-Scl70 might not be reliable and valid due to fluctuations during follow-up, as mentioned above.
5. The authors presented only a 10% positivity rate for anti-Scl70. From previous study of SSc in Thailand, the proportion of patients with anti-Scl70 was reported around 70-80% [Int J Rheum Dis. 2020 Jul;23(7):945-957.]. Why was the prevalence of anti-Scl70 in this study much lower than previous large cohort among Thais?

Minor points

1. The term anti-topoisomerase I and anti-Scl70 were alternately used in the text. The authors should choose only 1 term and make it consistency to the whole manuscript.

Is the work clearly and accurately presented and does it cite the current literature?

Partly

If applicable, is the statistical analysis and its interpretation appropriate?

Yes

Are all the source data underlying the results available to ensure full reproducibility?

No source data required

Is the study design appropriate and is the work technically sound?

Yes

Are the conclusions drawn adequately supported by the results?

Partly

Are sufficient details of methods and analysis provided to allow replication by others?

Partly

Reviewer Expertise:

Internal Medicine and Rheumatology

I confirm that I have read this submission and believe that I have an appropriate level of expertise to confirm that it is of an acceptable scientific standard, however I have significant reservations, as outlined above.

# Response

**Published:** 2024-05-27

**Authors:** Pattarin Pirompanich

**Affiliations:** Medicine, Thammasat University, Klong Luang, Pathumthani, Thailand

Thank you very much for you kindly review. We really appreciate your response and revised our manuscript as you suggested.

**Abstract**

**Point 1:** Mean BMI and the proportion of patient with positive for anti-Scl70 should be mentioned in abstract because they were endpoints of the study.

**Response 1: ** We have added this data on Abstract (Page 2, Line 9-11).

**Point** **2:** This study did not investigate the cost-effectiveness of spirometry. Please revise the conclusion in abstract section and also in conclusion section.

**Response** **2: ** We have deleted this sentence from the abstract section and also in conclusion section.

**Background**

**Point 1:** The authors should mention why BMI was in your interested to be an endpoint of the study. What is/are the rationales for defining the association between BMI and spirometry parameters?

**Response 1:**

 BMI was included because it's a readily available measure of body composition that might influence lung function through various mechanisms. Understanding this relationship can provide insights into disease severity and potential risk factors for SSc patients.

 We have added this data on Background (Page 2, paragraph 2, line 8-9) (reference 6).

- Ishikawa C, Barbieri MA, Bettiol H, Bazo G, Ferraro AA, Vianna EO. Comparison of body composition parameters in the study of the association between body composition and pulmonary function. BMC Pulm Med. 2021;21(1):178.

**Methods**

**Point 1:** The diagnosis of systemic sclerosis is now using the 2013 ACR/EULAR Classification criteria not from 1980 ACR criteria.

**Response 1: ** Thank you for your value suggestion. We have already revised (Page 3, paragraph 2, line 2-3).

**Point** **2:** 128 out of 211 SSc patients underwent spirometry, so nearly 40% were not performed the spirometry. This may cause selection bias. Of those 40% who were not performed the test, they might be too tired to do the test or have difficulty to do the test for example air leak from mouth related to skin tightness of face, etc. Please clarify this point.

**Response** **2: ** We have acknowledged these on limitation (Page 8, paragraph 5, line 10-12).

**Point** **3:** Spirometry parameters and BMI can dynamically change or fluctuate over the course of follow-up, but anti-Scl70 is still be stable. Please clarify when of the spirometry test as well as BMI was picked up for analysis.

**Response** **3: ** In cases with multiple PFT results for a single patient, the earliest test conducted during the study period was employed to mitigate the impact of ongoing treatment and disease progression. BMI was obtained at the time of the PFT was done.

 We have added this data in Methods (Page 3, paragraph 2, line 6-9).

**Point** **4:** The association between spirometry and anti-Scl70 might not be reliable and valid due to fluctuations during follow-up, as mentioned above.

**Response** **4: ** While spirometry parameters and BMI can fluctuate over time, anti-Scl-70 is known for its relative stability. To minimize the impact of ongoing treatment and disease progression on lung function analysis, this study used the earliest PFT result for each participant (Page 3, paragraph 2, line 6-7).

 However, this approach might not fully capture the potential influence of anti-Scl-70 levels on spirometry, as anti-Scl-70 is considered stable. Future longitudinal studies with repeated measurements of both anti-Scl-70 and spirometry parameters could provide a more comprehensive understanding of the interplay between these factors and their impact on lung function in SSc patients.

 We have acknowledged these on limitation (Page 8, paragraph 5, line 1-4).

 Further studies are required to evaluate comprehensive data on disease duration to gain a more understanding of the disease course and its impact on SSc patients.

 We have added this data in Conclusion (Page 8, line 4-5).

**Point** **5:** The authors presented only a 10% positivity rate for anti-Scl70. From previous study of SSc in Thailand, the proportion of patients with anti-Scl70 was reported around 70-80% [Int J Rheum Dis. 2020 Jul;23(7):945-957.]. Why was the prevalence of anti-Scl70 in this study much lower than previous large cohort among Thais?

**Response** **5:**  Among the analyzed samples, 14 patients (10.9%) were positive for anti-topoisomerase antibodies, while 10 patients (7.8%) were negative. However, anti-topoisomerase antibody status was unreported for a large portion of the study group (n=104, 81.3%) due to limitations in data availability. This high percentage of missing data **precludes definitive conclusions about the overall prevalence of anti-Scl-70 in our cohort and hinders comparisons with previous studies that likely had more complete data.**

**We **have added this data in Results (Page 3, paragraph 1, line 2-4).

**Minor point**

**Point** **1:** The term anti-topoisomerase I and anti-Scl70 were alternately used in the text. The authors should choose only 1 term and make it consistency to the whole manuscript.

**Response** **1: ** We revised throughout the manuscript.

**Additional revision: **Because the interest of brevity, we delete some wordings in Abstract and the total wording is 262 words.
